# A Tri-Satellite Interference Source Localization Method for Eliminating Mirrored Location

**DOI:** 10.3390/s21134483

**Published:** 2021-06-30

**Authors:** Lihuan Huo, Rulong Bai, Man Jiang, Bing Chen, Jianfeng Chen, Penghui Huang, Guisheng Liao

**Affiliations:** 1 The 54th Research Institute of CETC, Shijiazhuang 050081, China; bai_CETC@163.com (R.B.); jiangman_sjz@163.com (M.J.); chenbin_cetc@163.com (B.C.); jianfeng_ah@163.com (J.C.); 2 Hebei Key Laboratory of Electromagnetic Spectrum Cognition and Control (ESCC), Shijiazhuang 050081, China; 3 School of Electronic Information and Electrical Engineering, Shanghai Jiao Tong University, Shanghai 200240, China; huangpenghui@sjtu.edu.cn; 4 National Laboratory of Radar Signal Processing, Xidian University, Xi’an 710071, China; liaogs@xidian.edu.cn

**Keywords:** tri-satellite localization, interference source localization, cross-ambiguity function

## Abstract

With the increase in satellite communication interference, the tri-satellite time difference of arrival (TDOA) localization technique, which is an effective method to determine the location of the interference using sensors or antennas, has been developed rapidly. The location of the interference source is determined through the intersection of the TDOA lines of position (LOP). However, when the two TDOA LOP have two mirrored intersection points, it is theoretically difficult to determine the real location. Aiming at this problem, a method for eliminating mirrored location based on multiple moment TDOA is proposed in this paper. First, the TDOA results are measured at multiple moments using the cross-ambiguity function (CAF), and the localization equation set is established based on the World Geodetic System (WGS)-84 earth ellipsoid model. Then, the initial location result can be obtained by solving the equation set through the Newton iteration method. Finally, the high-precision location result after eliminating the mirrored location is obtained after the single moment localization based on the initial location. Simulation experiments and real measured data processing results verify the effectiveness of the proposed method. It still has good robustness under the condition of large measurement errors and deviations from the prior initial values.

## 1. Introduction

With the rapid growth of satellite communication services, the interference signals received are also increasing. Satellite interference source localization technology [1,2], which can receive electromagnetic signals passively to locate the interference source, is the principal method of satellite anti-interference technologies. Aiming at the complex interference environment of satellite communication, several advanced technologies have been proposed to suppress the satellite interference [3,4,5] in recent years. Meanwhile, several satellite interference systems have been developed [6,7], represented by a “NOSS” series of a marine surveillance satellite developed by the US Navy, the Transmitter Location System (TLS) model 2000 developed by the Interferometric Inc., the SatID developed and operated by Defence Research Angency (DRA) of the UK, and the State Space Satellite Monitoring System (SSSMS) developed by the State Radio Regulation of China. The satellite interference source localization technology has the advantages of wide coverage, long operating distance, concealed reception, and strong survivability. Using the passive location, the satellite localization technology has strong survivability and concealed reception. The localization technology is based on the long baseline interferometry method for the satellites to realize wide area coverage. In addition, the detection of very weak signals is achieved through correlation techniques. It can locate interference source targets quickly and accurately, and has an important role and broad application prospects in both civil and military fields.

The current satellite interference source localization methods mainly use the time difference of arrival (TDOA) and the frequency difference of arrival (FDOA) measured by the signals received from the main satellite and the adjacent satellites to determine the location. The localization methods can be classified into analytical methods [8,9,10,11,12,13,14], optimized solutions [15,16,17,18,19,20], and intelligent algorithms [21,22,23] in recent years. The analytical methods derive the closed-form solution of the nonlinear localization equation set based on mathematical approximation and the intermediate variable. The nonlinear equations of localization are transformed into a set of linear equations, in which the closed-form solution can be derived. In the optimization techniques, the localization problem is reformulated as a convex optimization problem and can be solved based on the convex optimization algorithm. However, the local convergence may be produced in the optimization procedure. Considering the nonlinear characteristics of the location equations, the intelligent methods are proposed in recent years to deal with the grid search problem. Compared with the exhaustive method, the intelligent search algorithm has a higher search efficiency. However, when the need for the location accuracy increases, the complexity of the algorithm increases exponentially.

Compared with TDOA measurement, the FDOA measurement requires more reference stations for satellite ephemeris calibration. Therefore, the tri-satellite TDOA localization system has lower cost and more reliable location results. The tri-satellite TDOA localization method is based on two TDOA lines of position (LOP) for intersection positioning. Affected by the ephemeris of the satellite and the location of the interference source, the TDOA LOP have two intersection points at a certain moment, and it is difficult to distinguish the correct location from the mirrored location of the interference source. The research in the existing literature mainly focuses on the optimization of TDOA measurement [24,25] and the solution of localization equations [26,27]. For the mirrored location that appears in the location process, the correct location can only be obtained based on the prior knowledge of the interference location in the conventional methods. When the prior knowledge is missed, the mirrored location cannot be eliminated. Therefore, it is urgent to propose an effective and robust localization method in which the mirrored location results are eliminated.

Aiming to address the problems above, this paper proposes a method to eliminate mirrored locations in tri-satellite TDOA localization based on the TDOA measured at multiple moments. First, the TDOA measure model based on cross-ambiguity function (CAF) is established. Then, a multi-moment TDOA localization equation set is derived based on the World Geodetic System (WGS)-84 earth ellipsoid model. The location results near the correct location of the interference source is directly obtained through the TDOA measurement data at multiple moments using the Newton iteration method. Finally, the precise iterative localizations are obtained using the multi-moment location result as the initial value in the iterative localization moment by moment. This method can obtain more accurate location results on the basis of eliminating the mirrored location. At the same time, it still has good robustness under the conditions of large deviation of the prior initial value and large TDOA measurement error.

The paper is organized as follows: Section 2 introduces the TDOA/FDOA measure model based on the CAF for satellite localization. Section 3 provides the multiple-moment TDOA localization method. Section 4 provides the proposed tri-satellite localization method. In Section 5, simulations are performed to validate the performance of our methods as well as the real data result analysis. We conclude this paper in Section 6.

## 2. TDOA/FDOA Measure Model

**Figure 1:**
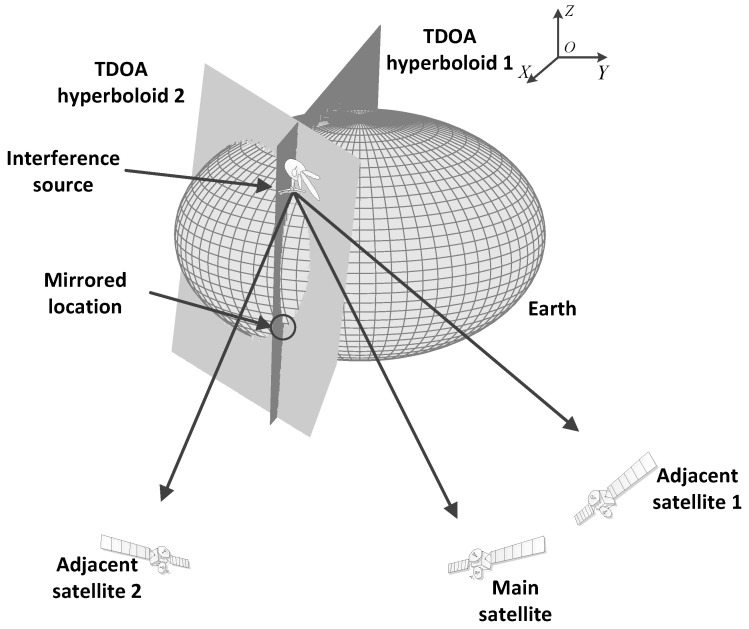
Configuration of the tri-satellite interference localization.

The model of the geodetic coordinate system is established as shown in Figure 1. The *X*-axis is pointing to the 0° longitude direction, the *Z*-axis is pointing to the center of the earth, and the *Y*-axis can be obtained according to the right-hand rule. It can be found that the main lobe of the interference transmitter is pointed towards the main satellite. At the same time, the two adjacent satellites can receive the sidelobe signal from the interference source.

Assume that the $s_{0}\left(t\right)$, $s_{1}\left(t\right)$ and $s_{2}\left(t\right)$ are the signal received by the main satellite, the first adjacent satellite and the second adjacent satellite from the interference source in time t. The received signals from the interference source in three satellites from can be expressed as:

(1) $$s_{0}\left(t\right)=s\left(t\right)+n_{0}\left(t\right)$$

(2) $$s_{1}\left(t\right)=A_{1}s\left(t-D_{1}\right)e^{j2\pi f_{d1}t}+n_{1}\left(t\right)$$

(3) $$s_{2}\left(t\right)=A_{2}s\left(t-D_{2}\right)e^{j2\pi f_{d2}t}+n_{2}\left(t\right)$$

where s(t) is the complex envelope of the signal received by the interference source in the second adjacent satellite, $A_{1}$ and $A_{2}$ are the amplitude ratio coefficients between the signals, $D_{1}$ and $D_{2}$ are the delay time of the signal transmission for the first adjacent satellite and the second adjacent satellite, $f_{d1}$ and $f_{d2}$ are the frequency differences, $n_{0}\left(t\right)$, $n_{1}\left(t\right)$, and $n_{2}\left(t\right)$ are observation noise indicated.

Since the received interference signal relayed by the adjacent satellite is the signal from the side lobe of the interference transmitter that points to the main satellite, its signal-to-noise ratio is generally low, even submerged in the noise. The communication terminal downlink signal relayed by the main satellite has a high signal-to-noise ratio generally. Aiming at this problem, the cross-correlation technique is used to realize the detection of weak sidelobe signals.

The second-order complex CAF of the received signal from the main and adjacent satellites is expressed as:

(4) $$A_{s_{0}s_{1}}^{\left(s\right)}\left(f,\tau \right)=Ae^{j2\pi f_{d1}t}A_{s}^{\left(s\right)}\left(f-f_{d1},\tau -D_{1}\right)$$

(5) $$A_{s_{0}s_{2}}^{\left(s\right)}\left(f,\tau \right)=Ae^{j2\pi f_{d2}t}A_{s}^{\left(s\right)}\left(f-f_{d2},\tau -D_{2}\right)$$

(6) $$A_{s}^{\left(s\right)}\left(f,\tau \right)=\frac{1}{\tau }\int_{0}^{T}s\left(t\right)s^{*}\left(t+\tau \right)e^{j2\pi ft}dt$$

According to the triangle inequality and the Schwartz inequality, we have:

(7) $$A_{s}^{\left(s\right)}\left(f,\tau \right)=A_{s}^{\left(s\right)}\left(0,0\right)$$

The estimation of the time delay ${\hat{D}}_{1}$ and ${\hat{D}}_{2}$ and the frequency difference ${\hat{F}}_{d1}$ and ${\hat{F}}_{d2}$ can be obtained corresponding to the maximum value of CAF:

(8) $$\left({\hat{F}}_{d1},{\hat{D}}_{1}\right)=\arg\max\left\{\left|A_{s_{0}s_{1}}^{\left(s\right)}\left(f,\tau \right)\right|\right\}$$

(9) $$\left({\hat{F}}_{d2},{\hat{D}}_{2}\right)=\arg\max\left\{\left|A_{s_{0}s_{2}}^{\left(s\right)}\left(f,\tau \right)\right|\right\}$$

## 3. Multi-Moment TDOA Localization Model

As shown in Figure 1, the hyperboloid surfaces can be obtained based on the TDOA. The first hyperboloid surface is formed based on the TDOA measured from the main satellite and the first adjacent satellite at some moment. The second hyperboloid surface is formed based on the TDOA measured from the main satellite and the second adjacent satellite at some moment. At each moment, the two TDOA measurements of the three satellites including the main satellite and two adjacent satellite can be measured to form the TDOA hyperboloid surface 1. Under the assumption of zero elevation of the interference source, the location result of the interference source is the intersection of the two TDOA hyperboloid surfaces and the earth ellipsoid. The intersection is the result of tri-satellite location. In the situation shown in Figure 1, it can be found that the TDOA surface intersects at a straight line, and the straight line intersects with the earth’s ellipsoid surface at two points. One point is the correct location of the interference source target, and the other is the mirrored position. Without any prior information, it is difficult to distinguish the correct location from the mirrored location only by the TDOA of a single moment.

**Figure 2:**
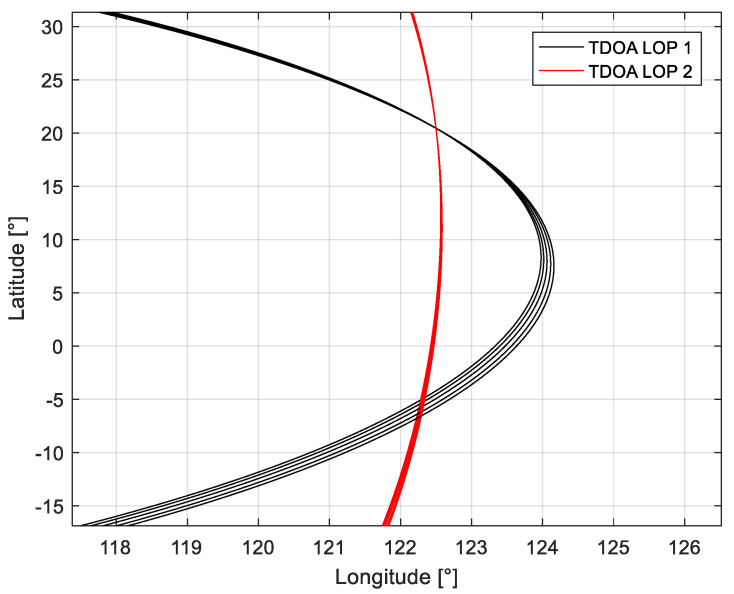
The intersection of the TDOA LOP in multiple moments.

The following Figure 2 shows the intersection of the TDOA LOP when there are mirrored points in tri-satellite localization. It can be found that the LOP at multiple times gradually converge to one point at the real position, with the TDOA LOP at the mirrored point position intersect being more divergent. Therefore, the TDOA measurement results at multiple times can be used for localization to determine the true location of the interference source.

Assuming that the coordinate of the interference source is (x,y,z), the coordinate of the main satellite at the first moment is $\left(x_{p0},y_{p0},z_{p0}\right)$, the coordinate of the first adjacent satellite is $\left(x_{p1},y_{p1},z_{p1}\right)$, and the second coordinate of the adjacent satellite is $\left(x_{p2},y_{p2},z_{p2}\right)$. The measured TDOA between the interference source and the first adjacent satellite and second adjacent satellite at the first moment are $\tau_{p1}$ and $\tau_{p2}$, respectively, which can be given by:

(10) $$\tau_{p1}=\left(r_{p0}-r_{p1}\right)/c$$

(11) $$\tau_{p2}=\left(r_{p0}-r_{p2}\right)/c$$

where c is the speed of light, $r_{p0}$ is the distance between the interference source of the main satellite, $r_{p1}$ is the distance between the interference source of the first adjacent satellite, $r_{p2}$ is the distance between the interference source of the second satellite, and p represents the p th moment.

According to the distance calculation method and the earth ellipsoid model, the tri-satellite localization equation set based on multiple moments can be given as follows:

(12) $$\left\{ \begin{array}{c} \sqrt{{\left(x-x_{11}\right)}^{2}+{\left(y-y_{11}\right)}^{2}+{\left(z-z_{11}\right)}^{2}}-\sqrt{{\left(x-x_{10}\right)}^{2}+{\left(y-y_{10}\right)}^{2}+{\left(z-z_{10}\right)}^{2}}=\tau_{11}\\ \sqrt{{\left(x-x_{12}\right)}^{2}+{\left(y-y_{12}\right)}^{2}+{\left(z-z_{12}\right)}^{2}}-\sqrt{{\left(x-x_{10}\right)}^{2}+{\left(y-y_{10}\right)}^{2}+{\left(z-z_{10}\right)}^{2}}=\tau_{12}\\ \vdots \\ \sqrt{{\left(x-x_{P1}\right)}^{2}+{\left(y-y_{P1}\right)}^{2}+{\left(z-z_{P1}\right)}^{2}}-\sqrt{{\left(x-x_{P0}\right)}^{2}+{\left(y-y_{P0}\right)}^{2}+{\left(z-z_{P0}\right)}^{2}}=\tau_{P1}\\ \sqrt{{\left(x-x_{P2}\right)}^{2}+{\left(y-y_{P2}\right)}^{2}+{\left(z-z_{P2}\right)}^{2}}-\sqrt{{\left(x-x_{P0}\right)}^{2}+{\left(y-y_{P0}\right)}^{2}+{\left(z-z_{P0}\right)}^{2}}=\tau_{P2}\\ \frac{x^{2}}{N^{2}}+\frac{y^{2}}{N^{2}}+\frac{z^{2}}{{\left[N\left(1-e^{2}\right)\right]}^{2}}=1 \end{array}\right.$$

where $N=a/\sqrt{1-e^{2}\sin^{2}{\hat{B}}_{k}}$ is the radius of the target local circle, a=6378137 m is the long axis of the earth, and $e^{2}=0.00669437999013$ is the square of the first eccentricity of the WGS-84 earth ellipsoid [28].

## 4. Improved Tri-Satellite Localization Method

Consider that it is difficult to directly solve the nonlinear localization equations formed by the measurement results at multiple moments. The Newton iterative method is used to solve it. The above equations can be expressed as follows:

(13) $$\left\{ \begin{array}{l} f_{11}=\sqrt{{\left(x-x_{11}\right)}^{2}+{\left(y-y_{11}\right)}^{2}+{\left(z-z_{11}\right)}^{2}}-\sqrt{{\left(x-x_{10}\right)}^{2}+{\left(y-y_{10}\right)}^{2}+{\left(z-z_{10}\right)}^{2}}-\tau_{11}\\ f_{12}=\sqrt{{\left(x-x_{12}\right)}^{2}+{\left(y-y_{12}\right)}^{2}+{\left(z-z_{12}\right)}^{2}}-\sqrt{{\left(x-x_{10}\right)}^{2}+{\left(y-y_{10}\right)}^{2}+{\left(z-z_{10}\right)}^{2}}-\tau_{12}\\ \;\vdots \\ f_{P1}=\sqrt{{\left(x-x_{P1}\right)}^{2}+{\left(y-y_{P1}\right)}^{2}+{\left(z-z_{P1}\right)}^{2}}-\sqrt{{\left(x-x_{P0}\right)}^{2}+{\left(y-y_{P0}\right)}^{2}+{\left(z-z_{P0}\right)}^{2}}-\tau_{P1}\\ f_{P2}=\sqrt{{\left(x-x_{P2}\right)}^{2}+{\left(y-y_{P2}\right)}^{2}+{\left(z-z_{P2}\right)}^{2}}-\sqrt{{\left(x-x_{P0}\right)}^{2}+{\left(y-y_{P0}\right)}^{2}+{\left(z-z_{P0}\right)}^{2}}-\tau_{P2}\\ f_{e}=\frac{x^{2}}{N^{2}}+\frac{y^{2}}{N^{2}}+\frac{z^{2}}{{\left[N\left(1-e^{2}\right)\right]}^{2}}-1,\;\mathrm{where}N=\frac{a}{\sqrt{1-e^{2}\sin^{2}{\hat{B}}_{k}}} \end{array}\right.$$

We assume that $F\left(x,y,z\right)={\left[\begin{array}{cccccc} f_{11} & f_{12} & \cdots & f_{P1} & f_{P2} & f_{e} \end{array}\right]}^{T}$, then the localization equation set can be formulated as:

(14) F(x,y,z)=0

The corresponding Hessian matrix is given as follows:

(15) $$F^{\prime }\left(x,y,z\right)=\left[\begin{array}{ccc} \frac{\partial f_{11}}{\partial x} & \frac{\partial f_{11}}{\partial y} & \frac{\partial f_{11}}{\partial z}\\ \frac{\partial f_{12}}{\partial x} & \frac{\partial f_{12}}{\partial y} & \frac{\partial f_{12}}{\partial z}\\ \vdots & \vdots & \vdots \\ \frac{\partial f_{P1}}{\partial x} & \frac{\partial f_{P1}}{\partial y} & \frac{\partial f_{P1}}{\partial z}\\ \frac{\partial f_{P2}}{\partial x} & \frac{\partial f_{P2}}{\partial y} & \frac{\partial f_{P2}}{\partial z}\\ \frac{\partial f_{e}}{\partial x} & \frac{\partial f_{e}}{\partial y} & \frac{\partial f_{e}}{\partial z} \end{array}\right]=\left[\begin{array}{ccc} \frac{x-x_{11}}{r_{11}}-\frac{x-x_{10}}{r_{10}} & \frac{y-y_{11}}{r_{11}}-\frac{y-y_{10}}{r_{10}} & \frac{z-z_{11}}{r_{11}}-\frac{z-z_{10}}{r_{10}}\\ \frac{x-x_{12}}{r_{12}}-\frac{x-x_{10}}{r_{10}} & \frac{y-y_{12}}{r_{12}}-\frac{y-y_{10}}{r_{10}} & \frac{z-z_{12}}{r_{12}}-\frac{z-z_{10}}{r_{10}}\\ \vdots & \vdots & \vdots \\ \frac{x-x_{P1}}{r_{P1}}-\frac{x-x_{P0}}{r_{P0}} & \frac{y-y_{P1}}{r_{P1}}-\frac{y-y_{P0}}{r_{P0}} & \frac{z-z_{P1}}{r_{P1}}-\frac{z-z_{P0}}{r_{P0}}\\ \frac{x-x_{P2}}{r_{P2}}-\frac{x-x_{P0}}{r_{P0}} & \frac{y-y_{P2}}{r_{P2}}-\frac{y-y_{P0}}{r_{P0}} & \frac{z-z_{P2}}{r_{P2}}-\frac{z-z_{P0}}{r_{P0}}\\ \frac{2x}{N^{2}} & \frac{2y}{N^{2}} & \frac{2z}{{\left[N\left(1-e^{2}\right)\right]}^{2}} \end{array}\right]$$

If the k th interference source coordinate in this iteration is $P^{k}=\left(x^{k},y^{k},z^{k}\right)$, it can be iterated according to the following formula:

(16) $$P^{k+1}=P^{k}-{\left[F^{\prime }\left(x^{k},y^{k},z^{k}\right)\right]}^{-1}F\left(x^{k},y^{k},z^{k}\right)$$

The process of the tri-satellite interference source localization method based on multiple moments is summarized as follows:

Step 1 Calculate the distance difference between the corresponding three satellites and the interference source $\left(r_{p1}^{}-r_{p0}^{}\right)$ and $\left(r_{p2}^{}-r_{p0}^{}\right)$ according to the TDOA at P moments;
Step 2 Initialization, set the number of iterations k=0, and the initial position of the interference source $P^{0}$;
Step 3 Calculate the Hessian matrix $F^{\prime }\left(x^{k},y^{k},z^{k}\right)$ in the process of this iteration, and obtain the estimation result of the interference source position $P^{k+1}$ in the next iteration according to formula (16);
Step 4 Define the position error $\delta =\sqrt{{\left(x^{k+1}-x^{k}\right)}^{2}+{\left(x^{k+1}-x^{k}\right)}^{2}+{\left(x^{k+1}-x^{k}\right)}^{2}}$ of the two iterations as the cost function in the iteration process. If it is less than a certain minimum threshold, stop the iteration and output the positioning result; otherwise, let k=k+1, and return to step 3 to continue the iteration.
Step 5 Precise localization moment by moment. The output location result is used as the initial value, and the single moment iteration method is used to obtain the precise value of the positioning result for the measurement result at each moment.

**Figure 3:**
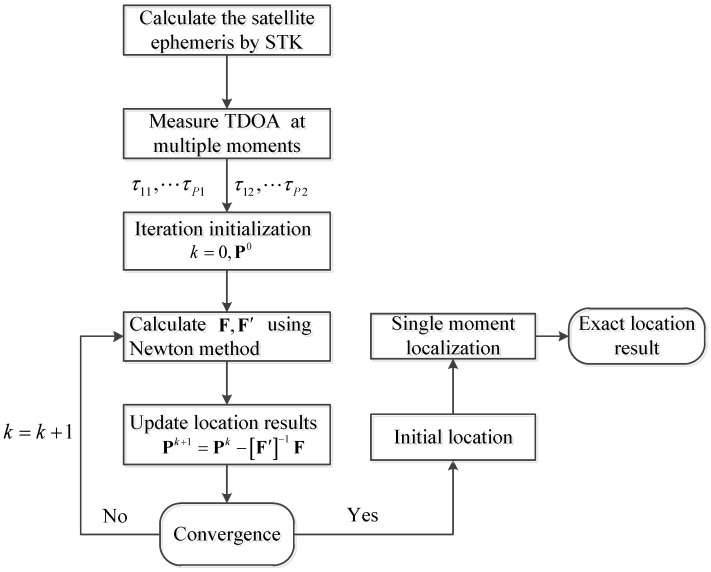
The flow chart of the tri-satellite interference localization.

The flow chart of the proposed tri-satellite interference localization is shown in Figure 3.

## 5. Results

In the following simulation, the number of localization moments in single-moment positioning is p=1 and the number of positioning moments in multi-moment positioning satisfies p>1. The proposed method is based on the multi-moment location results to remove the mirrored location. Then, the obtained location result near the correct location is used as the initial iteration for the single-moment localization method. The proposed method is the improved tri-satellite localization method based on the multi-moment TDOA localization.

### 5.1. Experiment on the Influence Factors of the Multi-Moment Localization

**Figure 4:**
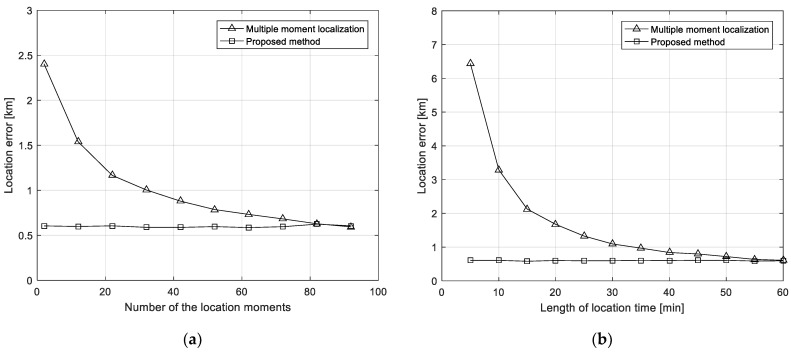
Experiments on the influence factors of location error. (**a**) Location errors versus the number of the location moments; (**b**) location error versus the length of location time.

The performance of multi-moment interference source localization is mainly related to the number of selected moments and the total length of time. The following will explore its change relationship through simulation experiments. The location of the interference source is 125° E, 30° N, and the TDOA measurement root mean square error is 0.5 μs. First, the total time is simulated for 20 min, and the relationship between multi-moment location method and the location error of the proposed method varies with the number of moments as shown in Figure 4a. It can be found that the performance of the multi-moment localization improves with the increase of the number of times, and continues to approach the proposed method. The location error of the proposed method is basically unchanged. Then, the relationship between multi-moment localization and the location error of the proposed method with the total time is simulated when the number of the moments is 10 as shown in Figure 4b. It can be found that the performance of the multi-time localization improves with the increase of the total time, and it continues to approach the proposed method. The location error of the proposed method also remains stable and can achieve high-precision and rapid localization.

### 5.2. Experiment on the Influence of Iterative Initial Location

**Figure 5:**
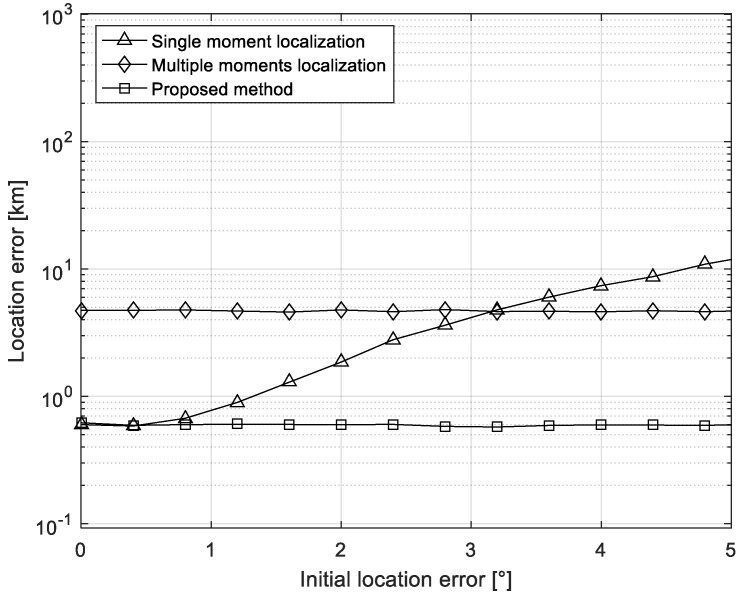
Localization errors versus the error of the initial location error.

The following simulation experiments are used to compare the effect of the initial location of the iteration on the performance of the algorithm. The location of the interference source is 125° E, 30° N, and the TDOA measurement errors are independent and identically distributed random variables distributed uniformly over [−0.5 μs,0.5 μs]. The number of Monte Carlo is 500. The time difference measured at three consecutive times is used to remove mirrored points for target localization. The time interval is five minutes, and the error of the initial iteration value is the root mean square error of the prior longitude and latitude deviation of the first iteration. Figure 5 shows the relationship between the location error of single-moment location, multi-moment moment, and the precise positioning method based on multi-moment measurement with the iterative initial value error when the iterative initial value error is between 0° and 5°. It can be found that, when the initial location error of the iteration increases gradually, the location error of the single-moment location degrades seriously. The multi-moment location method is basically not affected by the initial location error of the iteration, but with a poor performance compared with the single-moment method when the initial location error of the iteration is less than 3°. On the basis of multi-moment localization, the proposed method can obtain better location accuracy than the other two methods.

### 5.3. Experiment on the TDOA Measure Error

**Figure 6:**
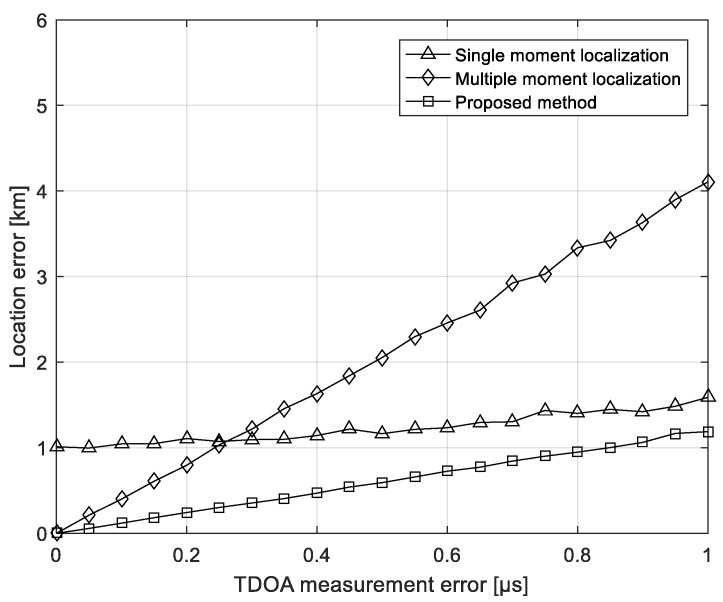
Localization errors versus the TDOA measure errors.

The following simulation experiments are used to compare the performance of the three methods over the TDOA measurement error. The location of the interference source is 125° E, 30° N, and the multi-moment location uses the TDOA measured at five consecutive times to remove mirrored points. The time interval is 4 min, and the root mean square of the initial location error of the iteration is 1°. The number of Monte Carlo is 500. It can be found in Figure 6 that, when the TDOA measurement error increases, the location error of the three methods increases. Among them, the single-moment location is mainly affected by the initial location error of the iteration when the TDOA measurement error is small, and it is not significantly affected by the TDOA measurement error. The multi-moment localization method is greatly affected by the time difference measurement error due to the method of solving the equations. The single-moment positioning method based on multi-moment measurement has good robustness when the time difference measurement error increases.

### 5.4. Real Data Result Analysis

**Figure 7:**
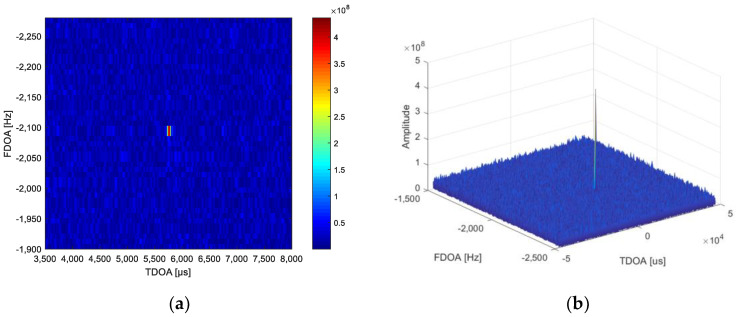

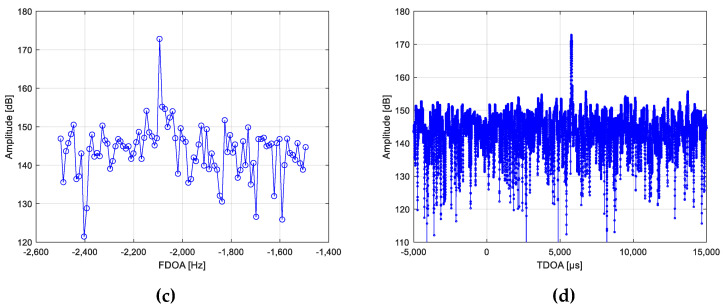
Results of the CAF processing using the real data in actual tests. (**a**) The CAF processing result in TDOA-FDOA plane; (**b**) the mesh of the CAF processing results in TDOA-FDOA; (**c**) the FDOA slice of maximum CAF amplitude; (**d**) the TDOA slice of maximum CAF amplitude.

In this section, the satellite real data processing results are presented to validate the effectiveness of the proposed algorithm. A signal from a cooperative interfering transmitter is illuminating the main satellite in synchronous orbit. The location of the interfering transmitter is [108.79° E, 32.32° N]. The two adjacent satellites can receive and relay the sidelobe signal of the interfering transmitter to the ground. This signal relayed from the three satellites is received by three C-band antennas, respectively. The CAF processing is used to measure the TDOA and FDOA of the main satellite and the adjacent satellites. The CAF processing results at a single moment are shown in Figure 7.

**Figure 8:**
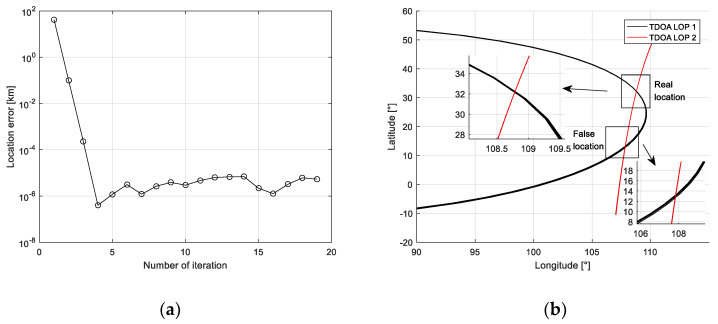
Results of the localization using the real data in actual test. (**a**) The location error versus the number of iterations; (**b**) description of what is contained in the second panel. Figures should be placed in the main text close to the first time they are cited. A caption on a single line should be centered.

After measuring the TDOA and FDOA of the received data from the satellite, the multi-moment localization and the proposed method are used to solve the location of the interference transmitter. It can be seen from the change of the cost function in the iterative process that the required results can be obtained by iterating 2–3 in Figure 8a. From the intersection of the time difference lines in Figure 8b, it can be seen that, due to the error in the time difference measurement, the time difference lines at the real point position at multiple times cannot completely intersect at one point. Compared with the real point position, the time difference line at the mirrored point is more divergent, so the method based on multi-time measurement can be used to remove the mirrored point.

The location results and location errors of the multiple-moment localization method and the proposed method in four moments are shown in Table 1. It can be found that the location errors vary with the location time, which is caused by the motion of the satellite. Meanwhile, the performance of localization of the proposed method is better than the multiple-moment location method with smaller location errors. The multi-time localization algorithm can locate near the real position of the radiation source target, but the accuracy is affected by the time difference measurement error, and it is difficult to obtain high localization accuracy. The proposed method can greatly improve the localization accuracy at multiple moments on the basis of removing mirrored points through subsequent point-by-point localization.

## 6. Conclusions

The tri-satellite TDOA localization uses the intersection of two TDOA LOP to determine the location of the interference source. However, if there are two intersection points between the two TDOA LOP, it is difficult to determine the correct point location. This paper proposes a mirrored point elimination method based on multi-moment measurement. This method uses the TDOA measurement data at multiple times to determine the area where the interference source is located in reality based on the convergence of the TDOA LOP at multiple moments. Then, the result is used as the initial value of the Newton iteration method to solve the single-time positioning equation. The location results with a high precision can be obtained after removing the mirrored points. The simulation experiment analyzes the influencing factors on the positioning performance, and verifies the robustness under the larger measurement error and the deviation of the prior initial value.

Meanwhile, the location accuracy of the proposed method is verified in real testing. Compared with the conventional method, the proposed method can effectively and quickly obtain higher accuracy. The ability of the algorithm is confirmed and can provide guidance on the real tri-satellite location projects.

## Figures and Tables

**Table 1:** Location results and location errors of the multiple time method and the proposed method.

Localization Method	Location Time	Location Result	Location Error
Multiple moment method	20 December 2020 14:29:54	108.7986° E 32.4250° N	11.66 km
	20 December 2020 15:29:54	108.7949° E 32.3725° N	5.84 km
	20 December 2020 16:29:54	108.8000° E 31.9776° N	37.93 km
	20 December 2020 17:29:54	108.8281° E 31.7897° N	58.86 km
Proposed method	20 December 2020 14:29:54	108.7899° E 32.2679° N	5.80 km
	20 December 2020 15:29:54	108.7937° E 32.3174° N	0.48 km
	20 December 2020 16:29:54	108.7938° E 32.2880° N	3.57 km
	20 December 2020 17:29:54	108.7938° E 32.3015° N	2.08 km

## Data Availability

Not applicable.
